# Biotic Threats to *Cycas micronesica* Continue to Expand to Complicate Conservation Decisions

**DOI:** 10.3390/insects11120888

**Published:** 2020-12-16

**Authors:** Benjamin E. Deloso, L. Irene Terry, Lee S. Yudin, Thomas E. Marler

**Affiliations:** 1 College of Natural and Applied Sciences, University of Guam, Mangilao, GU 96923, USA; delosob@triton.uog.edu (B.E.D.); lyudin@triton.uog.edu (L.S.Y.); 2 School of Biological Sciences, University of Utah, Salt Lake City, UT 84112, USA; irene.terry@utah.edu

**Keywords:** *Aulacaspis yasumatsui*, conservation science, Cycadaceae, *Erechthias*, invasion biology, *Luthrodes pandava*

## Abstract

**Simple Summary:**

Effective conservation of endangered plant species requires identifying their greatest threats to formulate management protocols. Invasive species are a result of global change and are a major threat to biodiversity. We used the island cycad *Cycas micronesica* K.D. Hill as a model that represents the global issues of conservation science and invasion biology. In Guam, several non-native insect invasions began in 2003 and have combined to threaten the island population of this cycad species. In this article, we summarize the history of reported invasions and the reported non-native insect herbivores that have recently increased the threat status. We also discuss the interactions among herbivores that threaten the sustainability of *C. micronesica* on the island of Guam.

**Abstract:**

Invasions of non-native species can threaten native biodiversity, and island ecosystems are ideal for studying these phenomena. In this article, first, we report on the invasive species that combine to threaten the island cycad *Cycas micronesica* by reviewing the history of previously reported invasions and providing an update of recent invasions. Then, we prioritize the threat status of each herbivore and the interactions among them. Plant damage was initiated in 2003─2005 by the non-native *Aulacaspis yasumatsui* Takagi armored scale, *Erechthias* sp. Meyrick leaf miner, and *Luthrodes pandava* Horsfield butterfly, which elicited unprecedented irruptions of the native *Acalolepta marianarum* Aurivillius stem borer and increased herbivory by feral pigs (*Sus scrofa* L.). The combined impact of these five consumers represents the greatest sustained threat to the cycad tree species. Mitigation of the damage caused by phytophagous non-native species is urgently needed to conserve this unique gymnosperm tree.

## 1. Introduction

The loss of biodiversity through extinction events has occurred since the beginning of life itself. The extinction of some organisms creates a crucial opportunity for the proliferation of other organisms that can exploit the changing environment. Biologists study this phenomenon and are particularly interested in the consequences of a relatively new extinction driver, that is, human activity during what is described as the Anthropocene epoch [1,2,3,4,5]. One unique facet of extinction during the Anthropocene is that biodiversity loss via extinction occurs without biodiversity replacement [6]. This may be partly due to the conversion of highly biodiverse localities into managed systems required to produce the crops and livestock needed to sustain the growing human population [7].

Conservation biology has evolved into a well-studied discipline that recognizes that anthropogenic mitigation actions must counteract this extinction crisis [8]. The interdisciplinary nature of conservation has resulted in the expansion of conservation biology into the discipline of conservation science, a term that better embraces the reality that social sciences and human landscapes must merge with the biology of natural systems to achieve conservation success [9].

The unprecedented volume of movement of species from their native range into new geographic locations is one of the main extinction drivers in the Anthropocene. Although this would appear to increase biodiversity, the negative impact of the new invasions on native organisms may actually decrease biodiversity [6]; therefore, due to the global influence of non-native organisms on native biodiversity, invasion biology has emerged as a bona fide discipline [10,11,12]. As this discipline continues to develop, the need to include multiple stakeholder groups such as humanities and social sciences has been recognized as a means of enabling successful outcomes [13]. Eradication of non-native species is most successful if organized efforts are initiated early in the invasion process. For this reason, employing non-professionals to monitor and report outbreaks of non-native species is a recent addition to the toolbox of invasion biology, a field that has been dubbed “citizen science” [14]. More recently, new frameworks have been created to organize the massive amount of data that has evolved from invasion biology research, which enables a more organized approach to compare case studies among global databases [15]. These developments allow biologists to study invasive species within their socio-economic or environmental impacts using globally relevant criteria.

The recent history of the island cycad known as *Cycas micronesica* provides a model case study that fully integrates conservation science with invasion biology. Improvements in the conservation of biodiversity require the support of adaptive management, both regionally and internationally. This can only be achieved if new knowledge from local research is shared to inform global conservation issues [16]. Here, we have updated the full list of known herbivore species that form a coalition of threats to Guam’s cycad tree species. Our results update the heavily researched case study of a culturally and ecologically important insular tree species that transitioned from being the most abundant tree on the island of Guam in 2002 [17] to being designated as endangered on the International Union for Conservation of Nature (IUCN) Red List in 2006 [18].

## 2. Materials and Methods

**Figure 1:**
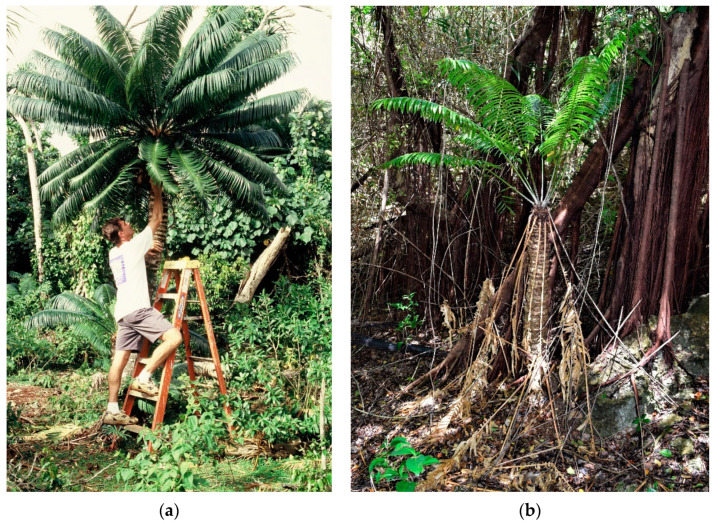
Guam’s population of *Cycas micronesica* trees were universally healthy prior to the 2003–2005 invasion of non-native insect herbivores. Very few of the remaining trees that persist in 2020 are healthy. (**a**) Collecting allometric data in December 1998 on a tree with no observable herbivory (T. Marler); (**b**) Gestalt appearance of many trees in September 2020. The white bases of the petioles are *Aulacaspis yasumatsui* infestations.

The identification of the *Aulacaspis yasumatsui* Takagi armored scale in Guam in 2003 [19,20] initiated our efforts to study the herbivore threats to Guam’s *C. micronesica* population. The *C. micronesica* trees exhibited no signs of leaf herbivory prior to this invasion (Figure 1a). Herein, first, we made one list of the various herbivore threats to this cycad species that have been previously reported in the literature. An exhaustive literature search on insect herbivory of *C. micronesica* was conducted in 2006 [19], and several other consequential insect herbivore threats have been described thereafter, but without a subsequent literature search. We used the single search word “*Cycas*” in Google Scholar, and limited the years to 2005–2020. The number of articles on this genus was constrained enough that no other search delimitations were needed. Then, we summarized the previously unreported insect species observed to consume *C. micronesica* tissue on Guam and Rota since 2006. Finally, in this article, we discuss the damage and interactions of the various herbivores observed from 2003–2020 to inform urgent conservation decisions that are needed for species recovery. These observations came from in situ locations of *C. micronesica*, in situ locations of the two primary insect herbivore threats, and common garden settings where *C. micronesica* is grown with numerous other *Cycas* species.

## 3. Results

Non-native herbivore damage has affected every *C. micronesica* tree in Guam over the past 17 years. Remnant populations of living trees struggle to survive the collective threat of several herbivores (Figure 1b).

### 3.1. Previously Reported Herbivores

Numerous phytophagous insects have been reported from *C. micronesica* trees in Guam. Two beetles and one Hemiptera were reported in 1942 (Table 1) [21,22,23]. A stink bug and soft scale were added to the list in 1946; a fruit fly, a planthopper, and three beetles were added in 1948 [24,25,26]. A nitidulid beetle was added in 1962 [27], then five Diaspididae species were added in 1966 and 1975 [28,29]. Specifics of the collection methods were missing from most of these reports; therefore assumptions that they were feeding on the *C. micronesica* tissue are unwarranted. Regardless, none of these individual species pose a threat to the host tree today, nor do they emerge as secondary threats that magnify plant damage caused by one of the primary threats.

Feeding on *C. micronesica* stem and leaf tissue by wild ungulates was reported to occur following a tropical cyclone [30]. Wild deer *Rusa marianna* Desmarest primarily fed on leaf tissue, and wild pig *Sus scrofa* L. fed on stem and leaf tissues.

Concerted efforts to understand the phytophagous insects that feed on *C. micronesica* began with the invasion of *A. yasumatsui* in 2003 (Table 1) [19]. Three other major threats to the species were quickly identified, including the *Cycas* specialist butterfly *Luthrodes pandava* Horsfield [31], the leaf miner *Erechthias* sp. Meyrick [19], and the stem borer *Acalolepta marianarum* Aurivillius [19]. Additionally, two Hemiptera, one Lepidoptera, and one Scarab beetle that cause minor damage were identified [19]. These efforts also led to the reporting of the cone borer *Anatrachyntis* sp. Meyrick as the first purported Lepidoptera pollinator species for any cycad [19].

The discovery of a probable Lepidoptera pollinator initiated an organized effort to understand the pollination system of *C. micronesica* fully. This led to identifying three nitidulid beetles that feed on *C. micronesica* microstrobili tissues (Table 1) [33]. These studies also confirmed that the *Anatrachyntis* and the three *Carpophilus* species were collected from sticky traps attached to megastrobili. Moreover, *C. micronesica* pollen was collected from the bodies of *Anatrachyntis* adults, further confirming their probable role as a native pollinator.

The continued observation of *C. micronesica* throughout Guam’s habitats led to the identification of two termites that feed on the stem or megasporophyll tissues (Table 1) [32]. Finally, after 13 years of damage to Guam’s *Cocos nucifera* L. population, the invasive *Oryctes rhinoceros* L. population exhibited a host shift to begin feeding on *C. micronesica* stem tissue [34].

### 3.2. Newly Reported Herbivores

The list of phytophagous animals confirmed to consume *C. micronesica* tissue has expanded since the last comprehensive review in 2006 (Table 2). We do not believe any of these newly added species act individually to substantially add to the threats to the host tree.

*Pseudaulacaspis cockerelli* Cooley is a widespread horticultural pest with an extensive host range. This armored scale has been considered the greatest scale problem in the Florida horticulture industry [35]. Male and female individuals look similar to those of *A. yasumatsui*, but the behavior of the two scale species differs in the initial infestation sites for Guam’s *C. micronesica* plants. First, *P. cockerelli* females preferentially infest the adaxial leaflet surfaces, but *A. yasumatsui* females preferentially infest the abaxial leaflet surfaces. Second, *P. cockerelli* males exhibit no preference for which leaflet surface is infested and form initial aggregations, but *A. yasumatsui* males preferentially infest the abaxial leaflet surfaces and do not aggregate. Third, in the absence of pesticide or biological control, *P. cockerelli* crawlers disperse and refrain from forming high-density infestations. Still, high-density *A. yasumatsui* infestations quickly develop at the initial infestation locations on a plant. Fourth, the extensive host range allows *P. cockerelli* to persist locally in the absence of a *C. micronesica* host plant, but *C. micronesica* is the only native host for *A. yasumatsui*. Fifth, the *P. cockerelli* infestations we have observed have been restricted to *C. micronesica* leaves, and *A. yasumatsui* readily infests *C. micronesica* leaves, stems, roots, megastrobili, microstrobili, and seeds.

An unidentified species of the large weevil genus *Myllocerus* sp. Schönherr has caused recurring damage on leaflets of *C. micronesica* and several other *Cycas* species in cultivation. The only in situ locality that we have observed *Myllocerus* damage to *C. micronesica* leaves was an area of occupancy adjacent to a golf course. The horticultural trees in the golf course likely sustained the localized weevil populations.

The termite *Coptotermes gestroi* Wasmann (synonym = *Coptotermes vastator* Light) is the third termite taxon that we have observed consuming *C. micronesica* stem tissue in habitat. The *C. micronesica* trees’ damage does not appear direct, as the feeding we observed was restricted to dead vascular tissue. However, the lowest strata of the infested trees’ structural integrity are compromised by the termite activity, which increases the plant population’s threat by reducing the tree’s ability to withstand tropical cyclone force winds.

*Hermetia illucens* L. larvae were observed consuming *C. micronesica* sarcotesta tissue from seeds heavily infested with *A. yasumatsui*. This insect’s primary larval food is detritus or feces, including in Guam [36]; therefore, the herbivory of tissue on living seeds was unexpected. We have never seen this herbivory on healthy seeds, so this herbivory has been restricted to damaged seeds with a history of *A. yasumatsui* infestations on the seed integument.

*Rattus rattus* L. subspecies *diardii* were observed on numerous occasions on the island of Rota, feeding on sarcotesta tissue of mature *C. micronesica* seeds. The seeds were still attached to living, intact megasporophylls in every case.

The cortex parenchyma of fallen *C. micronesica* stems was observed being consumed by the hermit crab *Coenobita* sp. Latreille on numerous occasions. An open wound often results in exposed cortex parenchyma when pigs begin to feed on standing, living *C. micronesica* stems. This crab is a secondary herbivore of the exposed parenchyma tissue after the initial pig damage.

The mollusc *Satsuma* sp. Adams was observed feeding on young expanding *C. micronesica* leaves. This snail herbivory was a one-time observation.

The orders Coleoptera and Hemiptera contained the greatest number of families and species of *C. micronesica* herbivores (Table 1 and Table 2). The number of species was similar for the two orders, but Coleoptera family richness was 1.6-fold greater than that of Hemiptera.

## 4. Discussion

### 4.1. Ranking of Individual Herbivores

Our sustained direct observations among Guam’s *C. micronesica* areas of occupancy and the urban landscapes have enabled an arbitration of the severity of each of the biotic threats to this unique native tree species. The plant organs consumed, the temporal versus chronic nature of the threat, and the lethality of damage contrast sharply among the herbivores. Over the past 17 years, our collective observations indicate that mitigation of these biotic threats comprises the only acute conservation need for reversing the plant mortality.

#### 4.1.1. *Aulacaspis yasumatsui*

Most of the historical *C. micronesica* mortality on the islands of Guam and Rota has been a result of *A. yasumatsui.* This armored scale was absent from both islands until 2003 when it was identified on Guam in a single urban landscape, then on Rota in a single outbreak site in 2007. The scale rapidly spread throughout both islands because of the population of *C. micronesica* and the widespread use of *Cycas revoluta* Thunb. trees in the urban environments on both islands. Direct observations of efficacious biological control within the native range of this scale and damage comparisons among many *Cycas* species within a common garden setting indicate the damage of *A. yasumatsui* to *C. micronesica* in Guam and Rota is due to the lack of biological control within these insular invaded habitats [37].

In the absence of adequate biological control, *A. yasumatsui* is by far the greatest ongoing threat to these insular *C. micronesica* populations for numerous reasons. It is the only *C. micronesica* herbivore on Guam and Rota that is lethal when acting alone. This sap-feeder is also the only herbivore that feeds on every plant organ (Table 1). More importantly, there is no temporal constraint on initial infestation success. The original crawlers that find a new plant can begin feeding and regenerating on any soft organ surface. A leaf’s risk is the same regardless of an age span of days or years. The base of the permanent stem of a tree that is centuries in age is at risk on par with a microstrobilus that emerges from the stem apex and completes its function in two months. Many Hemiptera insects pose a threat to the host plant by vectoring pathogens, but *A. yasumatsui* kills its host by the relentless depletion of non-structural resources and killing of newly emerging leaves before they can become a net carbon source organ [38].

The scale predator *Rhyzobius lophanthae* Blaisdell (Coleoptera: Coccinellidae) was released in Guam in 2005 [31]. We have identified three limitations to the predatory success of this animal. First, the *A. yasumatsui* individuals are so small that they can infest locations on *Cycas* organ surfaces that the relatively large predator cannot access [39,40]. Second, the predator tends to avoid the plants’ lowest stratum for unknown reasons such that *A. yasumatsui* infestations are uncontrolled on leaf, stem, and root surfaces near the ground [41]. Third, the olfactory signals from *A. yasumatsui* are less attractive to the predator when the infested leaves are from seedlings rather than adults [42]. These and other phenomena have limited this predator’s success in protecting the tree host from *A. yasumatsui* damage.

Numerous introductions and releases of *Coccobius fulvus* Compere & Annecke (Hymenoptera: Aphelinidae) and *Aphytis lingnanensis* Compere (Hymenoptera: Aphelinidae) parasitoids have been conducted in Guam to control *A. yasumatsui*, and to date we have been unable to verify that these animals have established; however, a fortuitous establishment of the scale parasitoid *Arrhenophagus chionaspidis* Aurivillius (Hymenoptera: Encyrtidae) was observed in 2013. This parasitoid avoids female armored scales, and preferentially parasitizes male armored scales; therefore, the parasitoid’s effectiveness is limited by this behavior.

#### 4.1.2. *Luthrodes pandava*

The *Cycas* specialist *L. pandava* does not pose a lethal threat when it is the only herbivore, but general plant vigor is damaged. This butterfly was in the Northern Mariana Islands of Rota, Saipan, and Tinian for many years before being identified in one northern Guam locality in 2005. The population spread throughout Guam along with *A. yasumatsui*, with both insects often entering new localities at the same time. The fact that the larvae require young, expanding tissue is both a limitation to the herbivore’s population success and why the herbivory is so damaging to plant vigor. The larvae require leaf tissue for greatest performance, but we have observed larvae feeding on young cataphyll, megasporophyll, and microstrobili tissue when no expanding leaves are available. All *Cycas* species grow with ephemeral flushes of leaves separated by quiescent periods that may last more than one year. The entire expansion phase of *C. micronesica* leaves is about 30 d [43]. If a gravid female *L. pandava* adult finds a leaf in the first week of expansion, the larvae will consume the entire leaf. If a gravid female adult finds a leaf in the second week of expansion, leaflets may be partially or entirely consumed, but rachis and petiole tissue is not consumed. Ovipositions that occur after two weeks may lead to partial leaflet herbivory but not complete leaflet consumption.

These behavioral phenomena of the host and this butterfly herbivore cause highly heterogeneous spatiotemporal herbivory levels within areas of occupancy where *C. micronesica* and *L. pandava* co-exist. In a common garden setting, *C. micronesica* is among the most damaged of *Cycas* species by this butterfly [44]. The speed of leaf expansion and maturation among *Cycas* species is one plant trait that determines resistance to *L. pandava* herbivory [45]. Leaf maturation of *C. micronesica* is slow compared to *Cycas* species that are resistant to this specialist herbivore. Damage to *C. micronesica* in Guam and Rota is primarily due to the host tree’s lack of resistance [37,44].

#### 4.1.3. *Erechthias*

**Figure 2:**
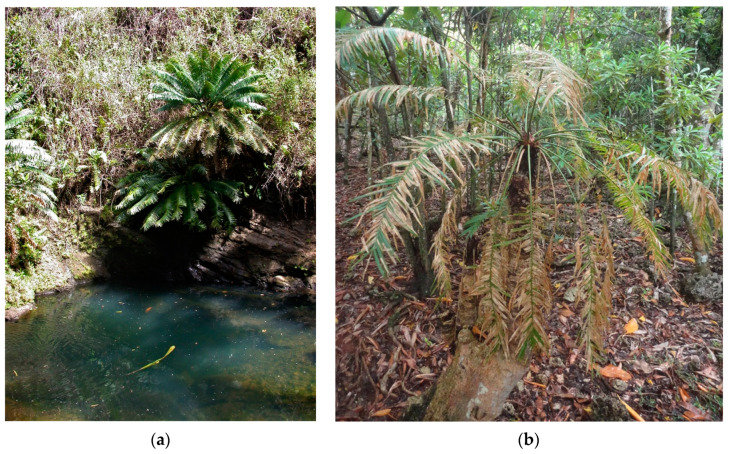
*Erechthias* leaf miner herbivory is restricted to the oldest of *Cycas micronesica* leaves. (**a**) Many of the youngest leaves exhibit no leaf miner damage in this riparian Guam habitat in December 2004; (**b**) 100% of the leaves on some trees exhibit leaf miner damage in June 2020.

The omnipresence of *Erechthias* throughout Guam causes this leaf miner to be the most damaging of the secondary threats. This leaf herbivore cannot kill the host tree when it is the only herbivore present, but general plant vigor is damaged. The initial outbreak was observed in one locality in southern Guam in 2003. To date, this leaf miner has not been observed on Rota. In relatively healthy trees, all of the youngest *C. micronesica* leaves are undamaged by this leaf miner (Figure 2a). For the first time since 2003, numerous Guam trees exhibit *Erechthias* damage on some leaflets on 100% of the leaves in 2020 (Figure 2b). This unprecedented level of leaf damage does not occur because the animal has exhibited a behavioral change to oviposit in younger leaves; instead, it is happening because the plants are producing leaf flush events less often, such that the youngest leaf cohort reaches the minimum age suitable for the leaf miner larvae. *Erechthias* damage is much less threatening to the host tree than *L. pandava* because herbivory is restricted to old leaves; however, the leaf miner damage is similar to *A. yasumatsui* damage, as every tree suffers from the chronic presence of the leaf miner population.

#### 4.1.4. *Acalolepta marianarum*

The native stem borer *A. marianarum* is more damaging to individual trees than *Erechthias*, but less damaging at the population level because infestations are not omnipresent. Unlike the other major herbivore threats, this beetle does not attack healthy host trees. The guild of insect herbivores that comprise stem and bark borers provides a crucial ecological function within their native range by overwhelming unhealthy individuals to remove them from the host population [46]. The stressors that reduce host tree health in a manner that leads to increased stem borer damage may be abiotic, such as tropical cyclones [47], or biotic, such as herbivory by other herbivore guilds [48]. The extensive contributions of this native insect to *C. micronesica* plant mortality since 2005 are not a result of a behavior change of the insect; perhaps they result from the fact that every *C. micronesica* tree on Guam has been compromised in health due to the non-native insect herbivory.

**Figure 3:**
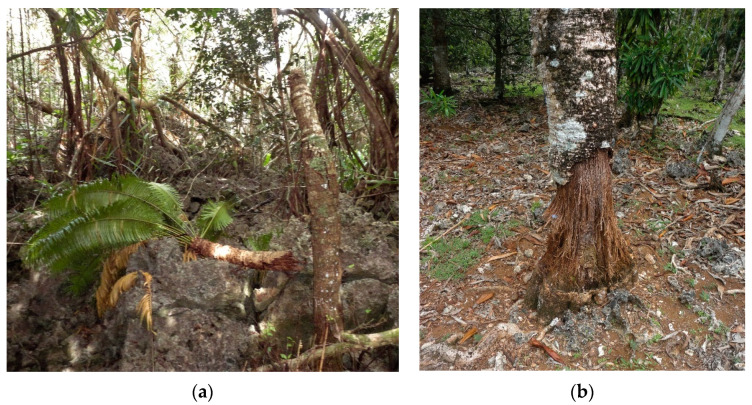
Changes in the behavior of some established herbivores have emerged in 2020. (**a**) *Cycas micronesica* stem breakage resulting from *Acalolepta marianarum* stem borer damage occurs in the absence of tropical cyclones for the first time. (**b**) Direct damage to standing living stems by *Sus scrofa* includes trees with no evidence of antecedent stem borer damage.

Two consequential changes in the relationship between *C. micronesica* and *A. marianarum* have occurred in 2020. First, the stratum that *A. marianarum* larvae damage *C. micronesica* stems has historically been restricted to 0.5–1.0 m [19]. For unknown reasons, this long-established stem borer behavior has changed in 2020, as we have observed *A. marianarum* stem damage as high as 3 m. Second, breakage of *C. micronesica* stems during the forces of tropical cyclone winds is increased by antecedent *A. marianarum* larval damage [49]. The breakage of stems during tropical cyclones was historically a combination of the compromised biomechanical strength caused by the stem herbivory and the lateral forces caused by the winds. This has changed in 2020, with numerous *C. micronesica* trees exhibiting breakage at the stratum of *A. marianarum* damage as a direct result of the herbivory alone and in the absence of tropical cyclone force winds (Figure 3a).

#### 4.1.5. *Sus scrofa*

*Sus scrofa* was introduced to Guam as a farm animal hundreds of years ago [50]. The wild pigs are a feral population that developed from these original farm animals. The nature of *C. micronesica* stems’ direct consumption has changed since the invasions of the non-native insects, dramatically increasing the threats of this ungulate to the tree population. Historically, pig herbivory had damaged no standing stems, but pigs sometimes ate broken stems dislodged from trees during tropical cyclones. Tropical cyclone damage to the *C. micronesica* trees increased following several years of non-native insect herbivory, and the first behavior change of the pig population was to completely consume every stem section that was detached during tropical cyclones. The second major behavior change of the pig population was to begin consuming cortex tissue on standing trees. Initially, the only trees that were eaten exhibited copious signs of *A. marianarum* larvae activity, and we believe this omnivore was attracted to the larvae as food. This behavior has shifted again in 2020. For the first time, the pig herbivory of standing *C. micronesica* trees can be observed on trees with no signs of *A. marianarum* activity (Figure 3b).

#### 4.1.6. *Oryctes rhinoceros*

The invasion of Guam by the scarab beetle *Oryctes rhinoceros* was first observed in 2007 [51]. Burrowing on stems of several non-native cycad species in a Guam garden was observed beginning in 2018, and burrowing on stems of *C. micronesica* throughout Guam was confirmed in 2020 [34]. The long-term threat that this new herbivore damage may exert on *C. micronesica* trees is not known. Burrowing occurs in the mature cataphyll complex immediately before a primary growth event. Subsequent male cones and leaves emerge distorted without the radial symmetry that characterizes *C. micronesica* organs’ primary growth; however, the cones and leaves can function despite the asymmetry. Moreover, when this beetle kills a stem’s apical meristem, the damage is lethal for palm species. In contrast, *de novo* development of adventitious buds occurs on *C. micronesica* stems, and these new buds enable new stem growth whenever the apical meristem is killed or removed from a tree [34].

#### 4.1.7. The Remainder of the Coalition

The many other confirmed herbivores do not individually pose a serious threat to the *C. micronesica* tree population. The possibility that one or more will exhibit a behavior change in the future remains a chronic threat. These herbivores contribute to *C. micronesica* destruction by interacting with one or more of the other major threats, and they add to the overall reduction in the vigor of the host plants.

### 4.2. Complicated Interactions Among the Threats

Non-native species may have benign or positive influences within their invaded territory. Distinguishing which invader species cause minimal effects from those that cause major effects is needed to formulate management decisions [52]. Moreover, one invader species’ direct negative influence on a native species is often studied in isolation, ignoring the probability that one invader interacts with other invader species [53]. These and other complicating issues demand that conservation decision-makers embrace a more holistic approach to determining invasive species’ influences on biodiversity by ensuring contextual evaluations are conducted by knowledgeable scientists [54]. This input of expert knowledge is critically important in conservation planning because of how complex the threats may be, the widespread lack of data, and the urgency with which mitigating actions need to be implemented [55].

The nexus of the long list of herbivores described in Table 1 and Table 2 is the unique tree *Cycas micronesica*. This gymnosperm tree survived for eons prior to human arrival to Guam and Rota. Still, now an ensemble of non-native herbivores and omnivores competes and abets each other to create a relentless assault on the host tree. One of the greatest challenges of developing a plan to control these threats is the extreme phylogenetic and guild differences among the consumers. Despite this difficulty, a focus on mitigating threats to plant species must embrace the complicated issues of covariation among threats and non-additivity among threats [56]. One threat may magnify the consequences of a second threat such that the imposition of two or more threats simultaneously may increase the overall damage to the growth and survival of a plant species. We described some of the consequential interactions of abiotic and biotic stressors to the *C. micronesica* population.

#### 4.2.1. Direct Competition

The *A. yasumatsui* and *L. pandava* herbivores exhibit behaviors that indicate direct competition, increases in damage from one occurs concomitantly with decreases in damage from the other [57]. We believe the combined damage of these two insects is non-additive, and the plant mortality has been magnified because of the concomitant herbivory of both insects. The joint damage of these two insects has been the major cause of the *C. micronesica* plants’ extensive mortality on Guam and Rota. If both of these invasive insects can be removed from Guam and Rota, we believe the extent of damage from the remaining threats would be minimal, and recovery of the threatened tree species would begin.

#### 4.2.2. Antecedent Damage Reduces Leaf Miners

The extent of *Erechthias* damage on Guam’s *C. micronesica* trees is controlled by antecedent *A. yasumatsui* or *L. pandava* damage. Irruptions of the armored scale or leaflet consumption by the butterfly preemptively damage the leaves before reaching the age that is suitable for the *Erechthias* oviposition. After the initial spread of the leaf miner throughout Guam habitats in 2003–2004, the damage declined when the initial *A. yasumatsui* outbreak occurred, then increased as the *R. lophanthae* predation of *A. yasumatsui* began partial control of the scale [57]. The nascent observations that *Erechthias* damage can be observed in 100% of the leaves on some *C. micronesica* trees in 2020 may mean the combined damage of *A. yasumatusi* and *L. pandava* has been declining in severity among the remaining living trees. Suppose the living trees were afforded some time to recover from the herbivory of all other consumers. In that case, we do not believe that sustained *Erechthias* damage would be a major threat to the tree population.

#### 4.2.3. Antecedent Damage Increases Stem Borers

The extent of *A. marianarum* damage is entirely under the control of stressors that reduce tree health. This native stem borer persisted with a compatible relationship with the *C. micronesica* population in Guam prior to the non-native insects’ invasions. Any abiotic or biotic stress or any combination of stressors can generate an irruption of *A. marianarum* by causing individual trees’ suboptimal health. The most productive conservation approach for minimizing *A. marianarum* damage is to mitigate the other threats to enable plant health recovery. This alone would mitigate the current *A. marianarum* threat.

#### 4.2.4. Herbivory Compromises Tropical Cyclone Resistance and Resilience

This gymnosperm tree serves as an example of a native species with resistance and resilience to a recurring native abiotic stress, tropical cyclones [30]. The recent insect invasions have removed much of this inherent resistance and resilience. Historically, catastrophic damage such as stem breakage or uprooting was minimal. When this did occur, the basal portion of a broken tree developed adventitious buds to resume stem growth. The stem sections that dislodged developed adventitious roots where the prostrate stem tissue was in contact with the soil surface [30,49]. This response to the tropical cyclone damage created two clones of the broken tree. Winching techniques were used to study the biomechanical strength of unhealthy *C. micronesica* stems [58]. The results verified that the stems of unhealthy trees were less able to resist horizontal displacement and predicted greater damage in future tropical cyclones due to the non-native herbivore damage. The prediction was confirmed by the incidence of Typhoon Dolphin in May 2015 [59]. Four international experts developed a management plan for in situ conservation of *C. micronesica* in northern Guam, which included the installation of guy wire anchoring on trees within management plots [60]. The efficacy of this engineered approach for anchoring was confirmed during Typhoon Dolphin as a conservation method to reverse the negative influence of the non-native insects on the tree’s resistance to wind damage [59].

#### 4.2.5. Urban-Natural Boundaries Not Respected

In situ conservation cannot be understood in isolation from urban horticulture because phytophagous insects do not respect urban-natural boundaries. This reality is magnified for Guam’s conservation efforts because resident biologists with the greatest level of species expertise are restricted from studying areas of occupancy within military installations even though the biotic threats to endangered species do not respect the boundaries between federal and non-federal lands. The role of horticulture in the study of island invasions is pertinent because the horticulture trade is responsible for many invasions [61,62]. Moreover, urban forests can serve as conduits through which non-native species expand their invasive range from one area of occupancy to a second area of occupancy. Ill-educated conservation practitioners may inadvertently serve as vectors of cryptic insects from natural systems to conservation nurseries and from urban forests to in situ locations if the practitioners do not understand the insects’ life history.

We describe two conservation examples that exemplify this phenomenon. First, an ex situ germplasm collection of Guam’s *C. micronesica* genotypes was established on Tinian island in 2008. This island is free of *A. yasumatsui*, so the conservation site has historically been safe from the most severe threat. This successful conservation project was managed by a species expert from 2008 with 1024 plants until 2018 when the collection was expanded to 1186 plants. The best practices for keeping the isolated site secure were ensured by training a crew residing on Tinian to meet the substantial workforce demands to maintain the large germplasm collection. This minimized the risk of inadvertent vectoring of *A. yasumatsui* from Guam to Tinian. A change in decision-makers within the funding agency occurred in 2018, which caused a shift in funding to a contractor without species expertise. The workforce to maintain the site began to be conducted by hazardous monthly trips of a maintenance crew from Guam to Tinian. The crawler stage of this scale often moves by hitchhiking on people [63], so conservation teams that understand this insect’s threats would have minimized travel from Guam to Tinian in continuation of the methods used from 2008–2018. If the federal permitting and funding agencies employed biologists with an understanding of the threats to the tree, the contractor would not have been allowed to use public funds to threaten the conservation site with these risky methods. Considerable mortality of the expensive germplasm has likely occurred since 2018 because the maintenance crew lacks training from a species expert. Second, *C. micronesica* trees have been rescued from Guam’s federal construction sites and transplanted to managed restoration sites. These anthropogenic actions increase stress among the transplanted trees in the managed recipient sites, generating a subsequent increase in vulnerability to *A. marianarum* damage. The restoration site then serves as a brood site for increased *A. marianarum* population to damage the in situ *C. micronesica* trees nearby the recipient site. The use of isolated locations far away from other in situ conspecific plants for recipient sites of transplanted native plants was proposed in 2017 as an approach to protect the surrounding native plant population from negative consequences of the restoration sites [37].

#### 4.2.6. Indirect Damage Worse than Direct Damage

The pachycaulous cycad stem is uniquely designed with a large primary thickening meristem and very few branches [64]. In fact, more than half of Guam’s female *C. micronesica* trees are unbranched [65]. Stem height growth is relatively slow, averaging about 3 cm per year for Guam’s population [66]. These traits reveal that stem breakage is highly damaging to a cycad plant’s continued health. The recent breakage of *C. micronesica* stems near the soil surface has been associated with termite damage. The recent breakage of *C. micronesica* stems at higher strata has been associated with *A. marianarum* damage. The loss of height by the tree impairs its competitive advantage among the sympatric trees, as hundreds of years of height growth may be lost on the single day of stem breakage. This case study provides a unique example where the insects’ direct herbivory may be less damaging than the indirect damage of stem breakage that results from the herbivory.

#### 4.2.7. The Two Wild Ungulates

The Philippine Deer (*Rusa marianna* Desmarest) was introduced to Guam in the 1700s as a wild hunting game [50,67]. This wild ungulate combined with the feral pig population to cause myriad ecological and conservation problems. The presence of deer in Guam’s forests is more damaging to general ecosystem function than the presence of pigs, with pigs providing an endozoochory function [68]. For *C. micronesica* species recovery, the opposite is true. The deer herbivory is restricted to leaves and megasporophylls and seasons with severe drought, which are rare in Guam. In contrast, the pig herbivory includes leaves, stems, and seeds and is chronic. The recent shift in pig behavior such that standing stems are eaten despite no signs of previous stem borer damage indicates that pig damage has emerged as a new acute threat to the tree species’ survival.

#### 4.2.8. Sequential Invasions Magnify Damage

In the absence of *A. yasumatsui* infestations, the seeds of *C. micronesica* are retained on the female trees for more than two years before they disperse individually [43,69]. The megasporophylls from which the seeds were dispersed usually remain intact on female trees after seed dispersal. Direct infestations of the armored scale on megastrobilus structures cause the base of the megasporophylls to break, with immature seeds being dispersed during the breakage. Old seeds that disperse individually are generally not consumed by pigs, but pigs consume the young seeds after dispersal. Our case study provides an example of how a non-native species (pigs) did not directly damage regeneration potential of a native tree species (*C. micronesica*) for hundreds of years until the invasion of a second non-native organism (*A. yasumatsui*) caused a change in the tree’s seed dispersal behavior.

#### 4.2.9. Protection from One Threat May Not Protect from a Second Threat

One factor that has protected Guam’s *C. micronesica* population from historical anthropogenic destruction is the steep topography of the coastal areas of occupancy. The relatively recent conversion of habitat to agriculture, military, and urban uses has decimated all native tree species’ populations on most of the northern Guam terrain, which is characterized as a tectonically uplifted plateau. The steep topography of the coastal regions has effectively protected *C. micronesica* from this historical and ongoing anthropogenic damage. This form of topographic protection of threatened gymnosperms has been identified as a benefit to biodiversity in other geographic regions [70]. The recent phytophagous insect invasions have effectively removed this long-established form of protection from anthropogenic damage, as the trees growing on unusable vertical cliff faces are as threatened by the herbivory as trees growing on flat terrain.

### 4.3. Recovery Coalitions

The influence of how coalitions of organisms influence conservation decisions is not restricted to planning the mitigation of coalitions of non-native threats. Coalitions of native organisms should also guide in defining targeted goals for threatened plant species recovery. Indeed, conservation of native species interactions should be a goal of all restoration plans, not just conserving the species [71]. For *C. micronesica*, the native interdependencies that have been disrupted by the recent anthropogenic activities include the host tree, the mutualist pollinator *Anatrachyntis*, the stem borer *A. marianarum*, the native mycorrhizae species that colonize the tree’s roots, and the native nitrogen-fixing *Nostoc* species that colonize the tree’s coralloid roots.

*Cycas micronesica* has been listed on the United States Endangered Species Act (ESA) for five years [72]. The direction of public funds for immediate conservation projects has focused on tree rescue from construction sites rather than the known threats to species survival [37]. The ultimate planning for *C. micronesica* recovery should embrace the need to conserve all native species that rely on the tree while eradicating or mitigating the non-native biotic threats.

#### 4.3.1. Protecting Pollinators

Conservation of the *Anatrachyntis* pollinators may pose the greatest dilemma for decision-makers. A biological control program for *A. yasumatsui* may use aggressive exploratory and permitting methods because there are no native armored scale species in the Mariana Islands that may suffer collateral damage. In contrast, a biological control program for the non-native *Erechthias* leaf miner carries great risk to the native *Anatrachyntis* pollinator population, as these two genera are closely related. Parasitoids may use cues from leaf-mining activity that are more challenging to find from mining activity [73], so a particular biological control organism that focuses on the leaf-mining stage may be available. Pursuing this would require the input of an expert in this form of biological control. The pollinator relies on frequent *C. micronesica* microstrobili production within an area of occupancy to provide brood sites to enable regeneration [74]. Less frequent production of smaller microstrobili has been occurring in recent years due to the severely damaged tree population. These plant behaviors carry the potential to reduce localized *Anatrachyntis* populations, which can reduce future pollination services. If the pollinator population crashes in some or all of the Guam and Rota areas of occupancy, *C. micronesica* populations’ potential to passively recover would be minimal. Some of the justification for the in situ conservation management plan in northern Guam [60] was to ensure the pollinator population had access to healthy *C. micronesica* trees within the management plots to sustain regeneration and ensure the pollinator remained viable for future *C. micronesica* species recovery.

#### 4.3.2. Passive Protection Best

The *A. marianarum* population in Guam is just as important for conservation as the *C. micronesica* population; therefore, attempts to implement the biological control of the stem borer to protect the tree population are unjustified. This native beetle poses no threat to the tree population in the absence of stressors such as the list of non-native herbivores in Table 1 and Table 2. Conservation projects should aim to minimize the other threats that compromise the trees’ health, which would remove *A. marianarum* as an acute threat to the tree. Inadvertent conservation of the stem borer in the coming years is ensured because the population of Guam’s unhealthy *C. micronesica* trees will sustain the beetle’s population until recovery of the plant population ensues.

#### 4.3.3. Root Symbionts

Conservation of the relationships among *C. micronesica* roots and the *Nostoc* and mycorrhizae mutualists should embrace a holistic approach. One of the greatest benefits of in situ conservation is that ecological interactions and mutualisms may be conserved even if they have not been studied and are not understood [75]. The Guam and Rota *C. micronesica Nostoc* and mycorrhizae mutualists have not been adequately studied. Efforts to remove all non-native plant and animal species from habitats may allow the native root symbionts to restore the native mutualisms passively. For example, the prevalent invasive trees that have altered the Mariana Islands’ landscapes have been shown to change chemistry and mineralization biology of soils compared with adjacent native trees [76,77]. For this reason, one of the most essential endeavors when constructing the in situ plots for conserving *C. micronesica* in northern Guam was exclusion of ungulates with fencing and removal of all non-native plant species [60], as *Carica papaya* L., *Leucaena leucocephala* (Lam.) de Wit, and *Passiflora suberosa* L. were present in high densities. Continued management of these plots must remain vigilant to ensure all non-native organisms remain excluded, enabling the passive conservation of the native edaphic mutualisms disrupted by the non-native ungulates and plants. This process may be slow, and the non-native plants’ sustained exclusion will be required for an extended period [78,79,80]. The transplantation of a native species to a restoration site within the native range of the species should ensure the recipient site’s natural conditions are suitable for accepting the transplants [81]. Many species transplant projects have been unsuccessful due to a lack of consideration that the recipient site should provide appropriate transplanted species conditions [82]. The primary recipient site being used for rescued *C. micronesica* trees in northern Guam is a degraded *Vitex parviflora* Juss. forest, a conservation approach that has been discouraged because of the altered soil biology in the degraded site that may damage the rescued trees’ health [77].

### 4.4. Future Research Needs

These complicated conservation issues indicate that species experts are needed to carefully monitor the interactions among the native species that rely on each other whenever conservation interventions are implemented to mitigate the non-native phytophagous insects that have invaded Guam. A loss of the functioning mutualisms among native species may precede the loss of any single organism [83]. These realities of the complexity of tropical landscapes have emerged as a limitation in tropical conservation science [84]. Numerous conservation projects designed without the input of knowledgeable scientists have consumed public funds and have failed or resulted in no sustainable longevity of effectiveness beyond the short-term funding cycles [85]. Evidence-based conservation should become the rule. This cannot occur when the decision-makers lack the subject area competence and the local scientists with the relevant knowledge do not have their knowledge accepted by those decision-makers [84,86].

Effective mitigation efforts require the identification of the primary threats by species experts. Regardless of funding and effort costs, preemptive actions will not be successful if conservation decisions fail to address the identified primary threats. Following are recommendations for continued research to support in situ *C. micronesica* conservation.

#### 4.4.1. Start Where It Starts

The *C. micronesica* plants on Guam and Rota were not threatened prior to 2003 when the invasions were initiated. This case study illuminates that the best approach for sustaining an unthreatened status of native tree species at risk of herbivory is to prevent the invasions before they occur. The 2020 level of threat and the number of non-native herbivores throughout the indigenous range of *C. micronesica* occur in the sequence Yap < Palau < Rota < Guam. Why are the *C. micronesica* populations in Yap and Palau persisting with no threats, but the populations in Guam and Rota suffering from acute threats? Numerous differences among these four geopolitical states may account for the disparity in contemporary threats, including volume of human travel, funding for adequate customs inspections, style of oversight by the federal governments, and conservation ethos of the residents [87,88]. The proficiency of the federal governments in each of these geopolitical states to accept and integrate international expert knowledge into conservation decisions may differ, creating an interesting case study to understand how federal policy-makers influence the successes or failures of conservation actions for a single threatened species that has a wide indigenous range [88].

#### 4.4.2. Successful Biological Control May Require Long-Term Commitments

Successful biological control of *A. yasumatsui* is achievable but will require an integrated multi-year plan for funding the cumulative process. We have collected parasitoids from *Cycas* leaves infested with *A. yasumatsui* in the Philippines and Thailand where the armored scale does not threaten its host *Cycas* trees, only to learn that they have not been described. The binomial is required to obtain an import permit, so these efficacious parasitoids cannot be introduced to Guam or Rota because they are unknown to science. An integrated funded program is needed to collect these parasitoids by cycad biologists, have them described by taxonomists, secure import permits with the new binomial, collect live animals by entomologists, then introduce and release them in Guam and Rota. This was proposed in 2017 as an urgent action for conserving *C. micronesica* [37].

The development of a biological control program for the *L. pandava* butterfly suffers from the probability of collateral damage to native butterflies. Biological control experts should be employed to determine if a highly specific biological control program is achievable.

#### 4.4.3. Pre-Existing Natural Stressors Must Be Integrated into Future Conservation Actions

Conservationists are unable to turn off the tropical cyclones that define life on Guam and Rota. The use of guy wire anchors for the trees within in situ conservation plots to protect the trees from catastrophic wind damage was a crucial component of the management plan developed by four international *C. micronesica* experts [60]. The value of the engineered anchors for conserving the trees has been confirmed [59]. More research into the biomechanical and allometric traits of *C. micronesica* stems and roots may improve management decisions and provide knowledge that can be used in other islands where threatened native tree species must contend with frequent tropical cyclones. This abiotic stress will persist in Guam and Rota, and conservation managers must integrate the stress into all decisions.

#### 4.4.4. Exploiting Fortuitous Developments

Guam’s *A. yasumatsui* infestations have become less widespread and less severe in the past two years (B. Deloso & T. Marler, unpublished). Many of Guam’s infestations have taken on the gestalt appearance of *A. yasumatsui* outbreaks within the native range of the scale, indicating newly efficacious biological control may have become established. The most recent parasitoid survey throughout Guam’s *C. micronesica* habitats was conducted in late 2017 and early 2018 and revealed *A. chionaspidis* remained the only parasitoid reared from *C. micronesica* leaves infested with *A. yasumatsui*. Studies to determine what appears to be fortuitously controlling *A. yasumatsui* on Guam in the past two years are urgently needed. We propose three possibilities. First, a new and unidentified parasitoid may have invaded Guam in recent years. Second, either *C. fulvus* or *A. lingnanensis* may have established following our many releases of these two parasitoids, but only recently irrupted to a population density such that effective biological control of *A. yasumatsui* was initiated. Third, the *A. chionaspidis* population known to be widespread in Guam may have become more efficacious in controlling the scale. Indeed, the combination of *A. yasumatsui* on *C. micronesica* in the Mariana Islands’ climate has never been available to this parasitoid, and perhaps these interacting factors combine to improve the parasitoid’s biological control abilities.

#### 4.4.5. One Size Does Not Fit All

The development of efforts to de-list *C. micronesica* from the ESA should be tailored for Guam and Rota separately, as the approach for species recovery may not be similar for the tree populations on the two islands. Due to lack of funding, we have not been able to observe the *C. micronesica* status on Rota in recent years. A *C. micronesica* species expert should be funded to assess Rota’s threat status, as many of the Guam threats were never identified on Rota during the ten years that we were actively studying the *C. micronesica* population on Rota prior to the loss of funding. A parasitoid survey should be conducted on Rota, and the parasitoids from Guam should be released on Rota if needed. More research on the frequent Rota *C. micronesica* seed gnawing by *R. rattus* may be useful for informing global island conservation efforts [89].

#### 4.4.6. Cascading Ecosystem Changes

The consequences of non-native insect herbivores on a native host tree are not restricted to mortality. We have reported that chronic herbivory of *C. micronesica* by these non-native insects has caused reductions in stem carbon dioxide efflux [90], has selectively killed more unbranched trees than branched trees to indicate more female trees have died than male trees [65], has compromised the success rate of adventitious root formation on stem cuttings [91], and has reduced the rate of tree height growth [66]. Additionally, we have reported substantial changes in leaf litter chemistry following herbivory in a manner that predicts increased decomposition rates [92,93]. The influences of these plant behavior changes on ecosystem traits need to be researched to fully understand how these non-native insects have influenced the host tree’s insular habitats.

#### 4.4.7. Species Experts Required

The observation that *O. rhinoceros* has exhibited a recent host shift from *Cocos nucifera* L. to *C. micronesica* throughout Guam [34] illuminates the need to have knowledgeable experts involved in all fieldwork associated with the conservation of this unique plant species. This nascent form of damage from a new pest went unnoticed until it was observable throughout the island primarily because the currently funded practitioners lack cycad expertise. The potential for another phytophagous insect invasion or development of an unprecedented novel interaction among the existing threats is substantial. Moreover, the original invasions of *A. yasumatsui* and *L. pandava* likely included a single subspecies from a single geographic origin. Both of these specialist insects have a wide indigenous range comprised of distinct subspecies from different geographic regions. The potential for a second invasion of a second subspecies remains as a potential threat from both of these insect species. If this were to occur, these two specialists’ genetic diversity would increase, which would subsequently increase the level of damage to the host tree. These developments will go unnoticed if species experts continue to be excluded from the conservation actions.

Continued research and conservation actions overseen by species experts are needed to fully understand this case study’s relevance to global conservation issues. An updated threat assessment by the IUCN is needed, as the last assessment was ten years ago [18]. A species recovery plan developed with expert knowledge is needed, as the addition of the species to the ESA was five years ago [72], and during those five years, 36% mortality of the 2015 cycad population has occurred [63]. This unique gymnosperm tree was the most abundant tree on Guam in 2002 [17]. For this reason, many of the historical ecosystem services provided by the taxon could be considered as those of a foundation species [94]. Based on the other organisms known to rely on the tree, these interdependencies can be used to consider the taxon as a keystone species [94]. The list of threats on each of the islands with areas of occupancy are dissimilar, illuminating *C. micronesica* as an excellent model for research designed to exploit the islands as natural experiments to more fully understand the role of fragmentation on biodiversity [94]. The funding of knowledgeable experts will be required for these opportunities to be fully exploited. How each of the major threats leads to plant mortality has been well-researched. Still, a full understanding of the threats’ interactions will require sustained direct observations by species experts.

## 5. Conclusions

We used a summation of the previously reported threats to the cycad known as *C. micronesica*. We reported an update on more threats that have not been previously reported to illuminate how single threats may interact to form a coalition of synergistic threats. As conservation decision-makers need multi-threat assessments such as ours to develop the most effective conservation actions [56], we use this well-researched case study as a model for informing conservation decisions of other threatened plant taxa [16]. Our report is useful for discussing the Anthropocene consequences and how the global disciplines of conservation science and invasion biology can be studied at the local level [1,2,3,4,5,6,7,8,9,10,11,12,13,14,15,16]. In the short term, mitigation of the phytophagous non-native species’ damage remains the only acute need to conserve this unique gymnosperm tree. For this purpose, the immediate focus should be on reversing the *A. yasumatsui*, *Erechthias*, *L. pandava*, and *S. scrofa* damage. We have estimated more than 70 years of demographic depth have been lost from Guam due to selective mortality of the smallest individuals [66]. If these threats are not mitigated, ongoing publicly funded projects designed to use seeds and cuttings to rescue the genetic diversity from construction sites are not advisable in the absence of a 70-year budget to protect the transplants from the ubiquitous non-native herbivores. Although much is known from a strong history of research, much of the available knowledge is not being used in conservation decisions, and much remains to be studied about the interactions among the threats.

## Figures and Tables

**Table 1:** Herbivores previously reported from *Cycas micronesica* on the island of Guam.

Order	Family	Species	Organ ^1^	Reference
Artiodactyla	Cervidae	*Rusa marianna* Desmarest	L,Me	[30] ^2^
Artiodactyla	Suidae	*Sus scrofa* L.	L,S,Se	[30]
Blattodea	Termitidae	*Nasutitermes* sp.	S	[32]
Blattodea	Termitidae	*Schedorhinotermes longirostris* Brauer	Me,S	[32]
Coleoptera	Anobiidae	*Dorcatomiella guamensis* Blair	U	[26]
Coleoptera	Cerambycidae	*Acalolepta marianarum* Aurivillius	S,Mi	[19] ^3^
Coleoptera	Chrysomelidae	*Phytorus lineolatus* Weise	NA	[26]
Coleoptera	Curculionidae	*Anaballus amplicollis* Fairmaire	NA	[23]
Coleoptera	Aderidae	*Euglenes bifossicollis* Blair	NA	[21]
Coleoptera	Mordellidae	*Mordellistena castanea* Ermisch	NA	[26]
Coleoptera	Nitidulidae	*Carpophilus dimidiatus* Fabricius	Mi+ ^4^	[33]
Coleoptera	Nitidulidae	*Carpophilus freeman* Dobson	Mi+	[33]
Coleoptera	Nitidulidae	*Carpophilus mutilatus* Erichson	S,Mi+	[27,33]
Coleoptera	Scarabaeidae	*Oryctes rhinoceros* L.	S	[34]
Coleoptera	Scarabaeidae	*Protaetia orientalis* Gory & Percheron	Mi	[19]
Diptera	Tephritidiae	*Cycasia oculata* Malloch	NA	[26]
Hemiptera	Aphrophoridae	*Lallemandana phalerata* Stål	NA	[22]
Hemiptera	Coccidae	*Ceroplastes ceriferus* Fabricius	L	[19]
Hemiptera	Coccidae	*Ceroplastes floridensis* Comstock	NA	[28]
Hemiptera	Coccidae	*Ceroplastes rubens* Maskell	NA	[24]
Hemiptera	Coccidae	*Saissetia coffeae* Walker	L	[19]
Hemiptera	Delphacidae	*Ugyops samoaensis* Muir	NA	[26]
Hemiptera	Diaspididae	*Aonidiella comperei* McKenzie	NA	[28]
Hemiptera	Diaspididae	*Aulacaspis yasumatsui* Takagi	L,R,S,Se,Me,Mi	[19]
Hemiptera	Diaspididae	*Lepidosaphes carolinensis* Beardsley	NA	[28]
Hemiptera	Diaspididae	*Lepidosaphes rubrovittatus* Cockerell	L	[29]
Hemiptera	Diaspididae	*Parlatoria proteus* Curtis	NA	[28]
Hemiptera	Pentatomidae	*Alciphron glaucus* Fabricius	NA	[25]
Lepidoptera	Cosmopterigidae	*Anatrachyntis* sp. Meyrick	Mi+	[19,33]
Lepidoptera	Lycaenidae	*Luthrodes pandava* Horsfield	L	[31] ^5^
Lepidoptera	Tineidae	*Dasyses rugosella* Stainton	S	[19]
Lepidoptera	Tineidae	*Erechthias* sp. Meyrick	L	[19]

^1^ L = leaf, Me = megastrobilus, Mi = microstrobilus, R = root, S = stem, Se = seed, NA = information not available ^2^ Reported as *Cervus mariannus* Desmarest. ^3^ Reported as *Dihammus marianarum* Aurivillius. ^4^ +indicates positive or neutral consequences to the host. ^5^ Reported as *Chilades pandava* Horsfield.

**Table 2:** Herbivores reported herein from *Cycas micronesica* on the island of Guam.

Order	Family	Species	Organ ^1^
Blattodea	Rhinotermitidae	*Coptotermes gestroi* Wasmann	S
Coleoptera	Curculionoidea	*Myllocerus* sp. Schönherr	L
Decapoda	Coenobitidae	*Coenobita* sp. Latreille	S
Diptera	Stratiomyidae	*Hermetia illucens* L.	Se
Hemiptera	Diaspididae	*Pseudaulacaspis cockerelli* Cooley	L
Rodentia	Muridae	*Rattus rattus* L.	Se
Stylommatophora	Camaenidae	*Satsuma* sp. Adams	L

^1^ L = leaf, S = stem, Se = seed.
